# Size regulation of the lateral organ initiation zone and its role in determining cotyledon number in conifers

**DOI:** 10.3389/fpls.2023.1166226

**Published:** 2023-05-17

**Authors:** David M. Holloway, Rebecca Saunders, Carol L. Wenzel

**Affiliations:** ^1^ Mathematics Department, British Columbia Institute of Technology, Burnaby, BC, Canada; ^2^ Biotechnology Department, British Columbia Institute of Technology, Burnaby, BC, Canada

**Keywords:** lateral organ, cotyledon, peripheral zone, apical meristem, morphogen gradient, spatial pattern, phyllotaxy, tissue size scaling

## Abstract

**Introduction:**

Unlike monocots and dicots, many conifers, particularly *Pinaceae*, form three or more cotyledons. These are arranged in a whorl, or ring, at a particular distance from the embryo tip, with cotyledons evenly spaced within the ring. The number of cotyledons, *n_c_*, varies substantially within species, both in clonal cultures and in seed embryos. *n_c_* variability reflects embryo size variability, with larger diameter embryos having higher *n_c_*. Correcting for growth during embryo development, we extract values for the whorl radius at each *n_c_*. This radius, corresponding to the spatial pattern of cotyledon differentiation factors, varies over three-fold for the naturally observed range of *n_c_*. The current work focuses on factors in the patterning mechanism that could produce such a broad variability in whorl radius. Molecularly, work in *Arabidopsis* has shown that the initiation zone for leaf primordia occurs at a minimum between inhibitor zones of HD-ZIP III at the shoot apical meristem (SAM) tip and KANADI (KAN) encircling this farther from the tip. PIN1-auxin dynamics within this uninhibited ring form auxin maxima, specifying primordia initiation sites. A similar mechanism is indicated in conifer embryos by effects on cotyledon formation with overexpression of HD-ZIP III inhibitors and by interference with PIN1-auxin patterning.

**Methods:**

We develop a mathematical model for HD-ZIP III/KAN spatial localization and use this to characterize the molecular regulation that could generate (a) the three-fold whorl radius variation (and associated *n_c_* variability) observed in conifer cotyledon development, and (b) the HD-ZIP III and KAN shifts induced experimentally in conifer embryos and in *Arabidopsis*.

**Results:**

This quantitative framework indicates the sensitivity of mechanism components for positioning lateral organs closer to or farther from the tip. Positional shifting is most readily driven by changes to the extent of upstream (meristematic) patterning and changes in HD-ZIP III/KAN mutual inhibition, and less efficiently driven by changes in upstream dosage or the activation of HD-ZIP III. Sharper expression boundaries can also be more resistant to shifting than shallower expression boundaries.

**Discussion:**

The strong variability seen in conifer *n_c_* (commonly from 2 to 10) may reflect a freer variation in regulatory interactions, whereas monocot (*n_c_* = 1) and dicot (*n_c_* = 2) development may require tighter control of such variation. These results provide direction for future quantitative experiments on the positional control of lateral organ initiation, and consequently on plant phyllotaxy and architecture.

## 1 Introduction

The positioning and arrangement of leaves and other lateral organs strongly influences plant phenotype and function. Lateral organs are commonly initiated in a peripheral zone around a growing tip such as the shoot apical meristem (SAM). The extent or position (radius or distance from the tip) of this initiation zone can be a critical factor in determining the type of phyllotaxy generated. Computational models demonstrated a switch from spiral to decussate (alternating) phyllotaxy upon a 33% increase in the radius of the SAM central zone (Jönsson et al., 2006); and Zhang et al. (2021) matched the changing phyllotaxy during *Gerbera* flower development through complex changes in the radius of what they termed the ‘active ring’. Experimental progress, in the model plant *Arabidopsis* in particular, is beginning to allow the manipulation of molecular pre-patterns, which affect the extent (size) of the lateral organ initiation zone (e.g. Caggiano et al., 2017).

**Figure 1:**
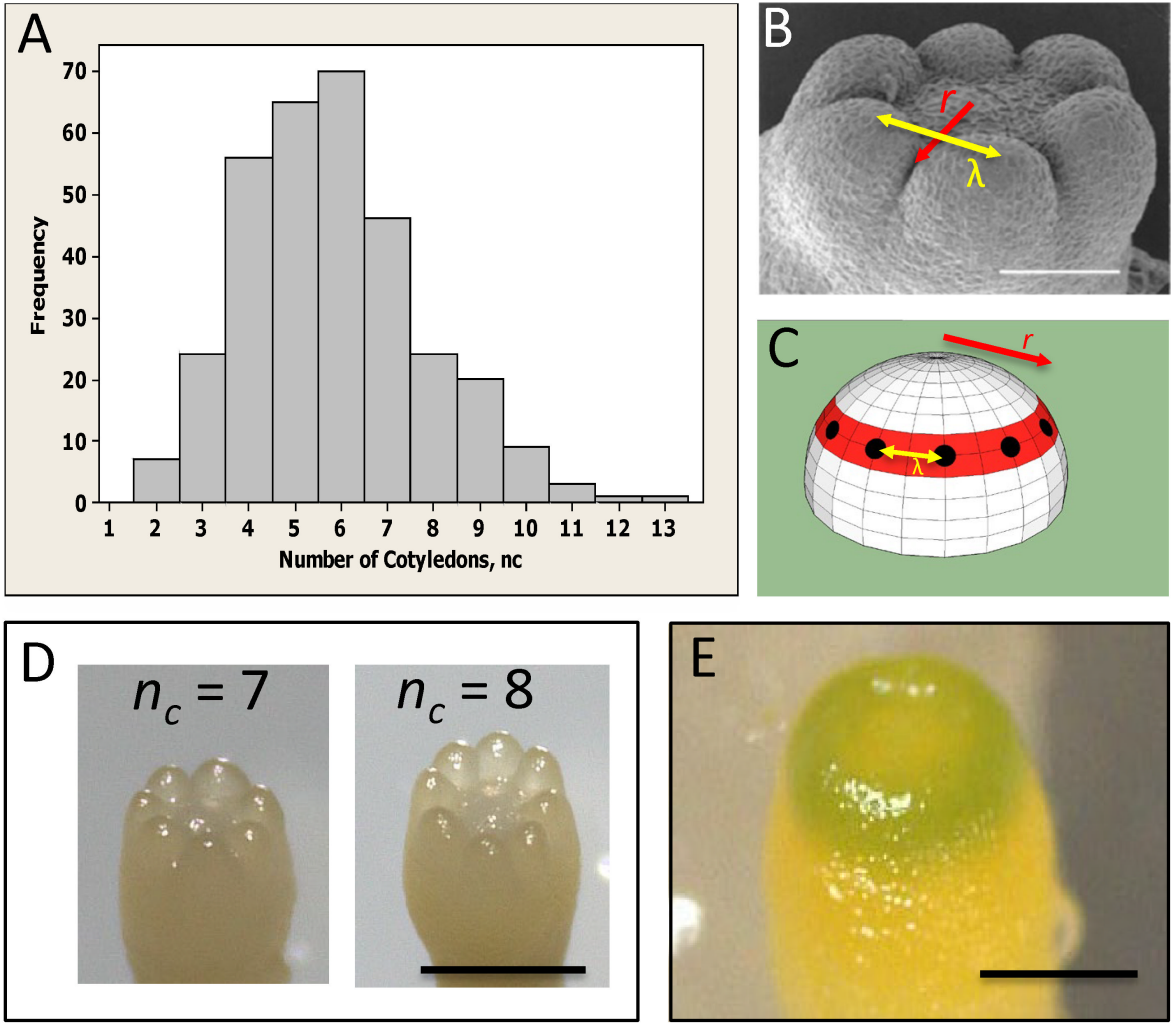
Whorled patterning and variable cotyledon number in conifers. **(A)** Cotyledon number, *n_c_*, is variable within a clonal culture of conifers (*Picea sitchensis* shown). **(B)** Cotyledons form in a single ring, or whorl, with a regular spacing λ (yellow arrow). **(C)** Cotyledon position is characterized by whorl radius *r* (red ring; red arrows in **(B, C)**) and intercotyledon spacing λ (yellow arrow): for a given λ, *r* determines how many cotyledons (black dots) fit in the whorl. **(D)** Examples of different cotyledon numbers in interior spruce embryos grown *in vitro* (*Picea glauca* - *Picea engelmannii* hybrid; courtesy of B. Wong). **(E)** A pre-cotyledon ring with early chlorophyll production (*Pseudotsuga gaussenii* grown *in vitro*; courtesy of L. Kong). **(A–C)** are adapted from Holloway et al. (2016) with permission. Scale bars = 0.2 mm **(B, E)**; ~0.5 mm **(D)**.

Conifer embryos show complex phyllotaxy at the earliest stages of lateral organ formation, during cotyledon development, presenting a unique system for investigating the role of the primordia initiation zone size in phyllotaxy. Unlike angiosperm monocots and dicots, many conifers (in particular *Pinaceae*) are polycotyledonous (Butts and Buchholz, 1940), with clonal cultures commonly displaying between 2 to 10 cotyledons (**Figure 1A**; von Aderkas, 2002; Harrison and von Aderkas, 2004; Holloway et al., 2016). While the number of cotyledons (*n_c_*) is variable, their spatial arrangement is highly conserved – always forming in a single ring or whorl, and showing a regular spacing (λ) between cotyledons in the ring (**Figure 1B**). This patterning is along two coordinates: (i) in distance from the tip, along which the radius *r* of the cotyledon ring is set (red, **Figures 1B, C**; referred to as P1, or Pattern 1, in Holloway et al., 2016; Holloway et al., 2018) and (ii) in a circumferential direction, along which the spacing between cotyledons (λ) within the ring is set (yellow arrows, **Figures 1B, C**; referred to as P2, or Pattern 2 in Holloway et al., 2016; Holloway et al., 2018). Correlation between embryo diameter and *n_c_* indicates that conifer *n_c_* variability reflects size variability between embryos (Harrison and von Aderkas, 2004; Holloway et al., 2016). For a particular P2 λ value (λ≈100 µm across several species measured in Harrison and von Aderkas, 2004; Holloway et al., 2016), the radius *r* of the P1 ring determines the number of cotyledons that can fit in, and therefore *n_c_* can provide a readout of initiation zone radius. P1 and P2 appear to have separable dependencies on polar auxin transport (PAT): treatment with the auxin-transport inhibitor N-1-naphthylphthalamic acid (NPA) interferes with the formation of separated cotyledons (P2), leaving cup-shaped (P1 pattern) embryos (Hakman et al., 2009; Holloway et al., 2016). This effect on P2 is consistent with the long established role of PINFORMED1 (PIN1) mediated PAT in establishing the auxin maxima at lateral organ initiation sites in the SAM (Kuhlemeier and Reinhardt, 2001; Reinhardt et al., 2003).

With respect to distance from the tip (P1 pattern), it has been shown in the *Arabidopsis* SAM that the auxin-PIN1 patterns specifying leaf initiation sites are constrained within a trough between a tip-high HD-ZIP III (specifically REVOLUTA, or REV, in *Arabidopsis*) domain and more basal KANADI (KAN) localization (Caggiano et al., 2017). HD-ZIP III and KAN are mutually inhibitory, with some of this interaction mediated by small RNAs, such as the HD-ZIP III inhibitor miR166, which emanates from the basal KAN domain (Caggiano et al., 2017). The HD-ZIP III *–* KAN interface remains important in subsequent leaf development, helping to define the adaxial/abaxial boundary of the growing blade (Kuhlemeier and Timmermans, 2016; Skopelitis et al., 2017; Heisler and Byrne, 2020).

HD-ZIP III *–* KAN patterning operates in embryos as well. In *Arabidopsis*, *HD-ZIP III* loss-of-function decreases the number of cotyledons, while gain-of-function increases the number of cotyledons (Prigge et al., 2005; Huang et al., 2014). Similarly in conifers, larch with overexpressed *miR166* have reduced *HD-ZIP III* expression and reduced formation of cotyledons (Li et al., 2016). *KAN* homologs are found across vascular plants (Zumajo-Cardona et al., 2019; all species BLASTed had GARP DNA-binding domains) including *Picea* conifers (El Kayal et al., 2011). In addition to the similar PAT-dependency of distinct primordia initiation sites in the SAM and in conifer embryos (P2 pattern), the HD-ZIP III - KAN mechanism regulating the distance from the tip to the organ initiation zone may be similar in *Arabidopsis* and in conifer embryos (P1 pattern).

**Figure 2:**
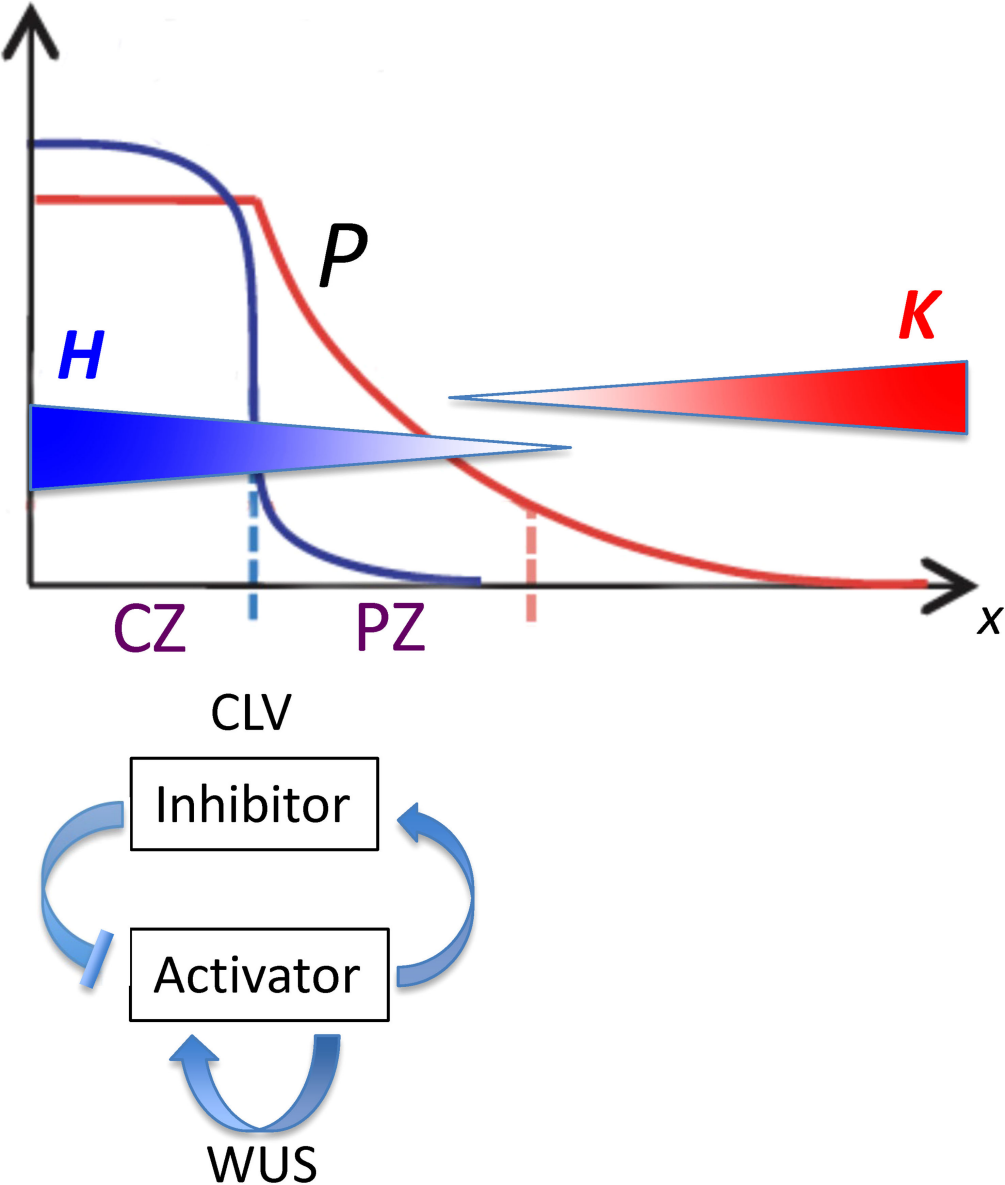
Spatial patterns governing the position of the lateral organ initiation zone plotted as position from the tip (*x*; horizontal axis) versus concentration (vertical axis). From Fujita et al, 2011, Figure 1; modified with permission. CLV-WUS dynamics early in development establish meristem zones: a central zone (CZ) characterized by a high WUS-induced activator (blue curve) becomes a source for morphogen *P* (red curve), which forms a gradient into the peripheral zone (PZ). Organs are initiated in the PZ between two inhibitory gradients of HD-ZIP III (*H*; blue gradient) and KAN (*K*; red gradient). The position from the tip of this initiation zone (dashed red line) depends on the characteristics of *P* as well as on the *H* and *K* dynamics. With reference to Eq. 4, *P_0_* is the maximum height of the red curve *P*; *s_e_* is the spatial extent of the flat part of *P*; and *B* is the curvature of the curved part of *P*.

We wanted to determine how factors in the HD-ZIP III *–* KAN dynamics might influence the positioning of the HD-ZIP III *–* KAN trough, and thereby the radius of the primordia initiation zone, impacting SAM phyllotaxy or *n_c_* in conifer embryos. For this, we used a quantitative model of HD-ZIP III *–* KAN regulation to characterize the influence of the following: (i) spatial patterning in upstream activators of HD-ZIP III; (ii) the activation dynamics of HD-ZIP III (e.g. Hill kinetics); and (iii) the mutual inhibition of HD-ZIP III and KAN or miR166. HD-ZIP III *–* KAN dynamics (ii, iii) are modelled on top of the pre-existing pattern (i), in particular the localization domains of meristem factors. Specifically, the current model starts from the positional information gradient (*P* or *Precursor*, **Figure 2**) generated by CLAVATA (CLV) - WUSCHEL (WUS) dynamics (for which, for example, see Fujita et al., 2011). While the model representation of HD-ZIP III (*H*) and KAN (*K*) likely generalizes multiple molecular factors, with potential differences in molecular identity across species, the quantitation of tip patterning allows us to characterize how pre-pattern, activation and mutual inhibition could contribute to the large range in conifer *n_c_*. From this, predictions can be made on the *n_c_*-variation that could be expected from future molecular perturbation experiments. The model also creates a framework for the positioning of the leaf initiation zone at the SAM, in particular how regulation of molecular pattern formation can affect phyllotaxy.

Larger conifer embryos have larger whorls with more cotyledons than smaller embryos (whorl radius approximately coincides with embryo radius; see **Figures 1B, D, E**). This indicates that the mechanism controlling the distance from the tip scales the P1 ring radius with embryo size. This is similar to a well-studied example of scaling in *Drosophila*, in which the maternal positional information gradient of the Bicoid (Bcd) protein (Driever and Nüsslein-Volhard, 1988) is longer in longer embryos, such that downstream genes are transcribed farther from the head (in absolute units, e.g. µm) in longer embryos than in shorter ones (Cheung et al., 2014; He et al., 2015; Wu et al., 2015). The differences in downstream expression positions, approximately 100 µm from short to long *Drosophila* embryos (Figure 6 of Cheung et al., 2014), are similar to differences in conifer whorl radius *r* from low *n_c_* to high *n_c_* (section 3.1). This may suggest similar limits in the scaling properties between the Bcd system and the HD-ZIP III – KAN positioning mechanism.

Unlike *Drosophila*, in which growth is negligible during Bcd scaling (Cheung et al., 2011), conifer embryos grow during cotyledon formation. This growth is insufficient, however, to produce the observed variation in whorl radius *r*. If, for example, the HD-ZIP III *–* KAN interface formed at a single consistent small *r* value (in µm), subsequent embryo growth is not fast enough to account for the observed variation in whorl radii: embryo diameter growth is commonly up to 20%/week (Figure 7 of Holloway et al., 2016), too slow to generate the observed 300% whorl radius variation (section 3.1) in the approximately 5 – 6 weeks from embryo induction to cotyledon morphogenesis *in vitro* (e.g. to the stage in **Figure 1B**). Additionally, embryos with a range of *n_c_* arise at approximately the same time in culture; there is no correlation between growth rates and *n_c_* (Holloway et al., 2016) and *n_c_* does not change during the growth of individual embryos. Combined with the HD-ZIP III, KAN and miR166 molecular effects, this suggests that *r*-variability is largely due to variation in molecular expression domains. We use growth-corrected whorl radius data (section 3.1) to focus on these molecular patterning effects.

We previously showed that a reaction-diffusion mechanism for P1 can robustly form a single ring over a three-fold range in radii (Holloway et al., 2018). Coupled to a P2 reaction-diffusion model, which locally catalyzed growth, this produced whorled cotyledon phyllotaxis. Two-stage P1-P2 control was shown to be particularly important for maintaining whorled phyllotaxy in large embryos. In that study, different tip sizes were input to the model to establish pattern robustness; the origin of whorl radius variability was not addressed. We now focus on this question: what, in the potential HD-ZIP III *–* KAN dynamics, can influence the conifer cotyledon whorl radius and generate its observed three-fold variation? More generally, how can HD-ZIP III *–* KAN dynamics regulate the radius of the lateral organ initiation zone and therefore influence phyllotactic pattern selection?

## 2 Materials and methods

### 2.1 Data

Somatic embryos were induced from interior spruce callus cultures (*Picea glauca* - *Picea engelmannii* hybrid, from the B.C. Tree Seed Centre). The basic medium (BM) contained the following: [(2.5 g/L Litvays salts, 0.1 g/L inositol, 14 mg/L FeSO_4_•7H_2_0, 19 mg/L Na_2_EDTA•2H_2_0, 20 g/L sucrose, 0.5 g/L glutamine, 1 mg/L thiamine HCl, 1 mg/L pyridoxine HCl, 5 mg/L nicotinic acid) adjusted to pH 5.6–5.8 and then 2 g/L phytogel was added prior to autoclaving]. Zygotic embryos were sterilized (10–15 s in 70% v/v EtOH, then 15 min in 15% v/v bleach, then 3 times sterile water) and plated for 2 wk on initiation medium [BM containing 2 mg/L 2,4-dichlorophenoxyacetic acid (2,4-D), 1 mg/L benzyl adenine (BA), 100 mg/L casein hydrolysate). Calli were subcultured biweekly on maintenance medium (BM medium containing 2 mg/L 2,4-D and 1 mg/L BA) for about 8 months. Calli were then subcultured biweekly on maturation medium (BM medium containing 16 mg/L abscisic acid and 0.2 mg/L indole-3-butyric acid) to induce somatic embryos. Measurements were taken of embryo diameter, cotyledon length and intercotyledon spacing on *n* = 237 embryos over the course of cotyledon development (from initiation through extension), from 7 to 16 weeks on maturation medium.

### 2.2 Mathematical model

A mathematical representation of the reaction and transport dynamics of a tip-localized factor (*H*; HD-ZIP III; e.g. REV in *Arabidopsis*) and its basal antagonist (*K*; e.g. KAN in *Arabidopsis*, or co-localized factors such as miR166) is used to characterize and quantify the regulatory factors that could contribute to positioning the whorl radius *r*. In addition to *H* and *K*, a precursor *P* is introduced to represent upstream patterning that could affect *H-K* positioning. For the SAM, *P* represents the size of the central zone (CZ) and its peripheral signalling (**Figure 2**), a pattern largely determined by CLV-WUS dynamics earlier in development (Clark et al., 1997; Brand et al., 2000; Jönsson et al., 2005; Fujita et al., 2011; Fujita and Kawaguchi, 2013; Klawe et al., 2020). *P* could also encompass effects from tip-localized factors such as auxin (Heisler and Byrne, 2020). The equations and terms, built from kinetic principles, are intended as representations of general processes, to guide future experiments and be refined by results.

HD-ZIP III and KAN are spatially patterned in the distance-from-the tip coordinate (i.e. along the lines of longitude, **Figure 1C**; apical-basal direction), and are roughly uniform in the circumferential coordinate (i.e. along lines of latitude, **Figure 1C**, at a particular apical-basal distance). Therefore, modelling is in one spatial dimension *x*, distance from the tip, over which *H* and *K* vary and on which the whorl radius *r* is specified. The model is solved on the interval 0< *x*< 3, corresponding to a domain of 0 (tip position) to 300 μm. Embryos flatten from a roughly hemispherical shape to a flattened tip during cotyledon formation (Nagata et al., 2013; Holloway et al., 2016; Holloway et al., 2018). At the flattened stage, the cotyledon whorl is approximately on a disk (**Figure 1B**), and radius *r* coincides with distance from the tip to the whorl along the surface. For the earlier non-flattened geometry, distance along the surface (i.e. meridional distance along a line of longitude, **Figure 1C**) is longer than *r* (horizontal distance from centre to whorl, **Figure 1C**), going to $\frac{\pi }{2}r$ at the hemispherical limit. Computational results are presented for the experimentally observed *r* range between 55 and ~180 μm (section 3.1). These positions correspond to distance from the tip *x* on the flattened tip; meridional *x*’s would be larger than *r* for a curved tip (e.g. 55< *r*< 180 corresponds to 56< *x*< 283 on a hemisphere of radius 180).

The model for *P*, *H* and *K* dynamics is:


(1) $$\frac{\partial P}{\partial t}=P_{0}-k_{P}P+D_{p}\frac{\partial^{2}P}{\partial x^{2}}$$

(2) $$\frac{\partial H}{\partial t}=prodH\left(\frac{P^{n_{H}}}{{\left(K_{A}\right)}^{n_{H}}+P^{n_{H}}}\right)\left(\frac{1}{1+inhH\cdot K}\right)-k_{H}H+D_{H}\frac{\partial^{2}H}{\partial x^{2}}$$

(3) $$\frac{\partial K}{\partial t}=prodK\left(\frac{1}{1+inhK\cdot H}\right)-k_{K}K+D_{K}\frac{\partial^{2}K}{\partial x^{2}}$$

All three species are synthesized, degraded (*k* terms), and allowed to move spatially (with diffusion coefficients *D_P_, D_H_, D_K_*). Parameter values are **Table 1**. Eqs. 1 – 3 were solved numerically with the *Matlab 2021* function *pdepe* on a spatial grid of 300 points (one per μm) to a steady state solution at *t* = 200 time units (i.e. run to twice the time at which patterns appear constant). Code is available at https://github.com/davidhollowaybc/lateral-organs file *pdePHKA.m*

**Table 1:** Model terms and values.

Parameter	Description	Value
***P***	Precursor concentration (meristem prepattern)	
*P_0_*	*P* production rate	100 within *s_e_*
*s_e_*	*P_0_* spatial extent	0.1
*k_P_*	*P* decay constant	1
*D_P_*	*P* diffusivity	0.5
*B* (Eq. 5)	*P* gradient exponential decay constant	** $\sqrt{k_{P}/D_{P}}$ **
*a* (Eq. 7)	*P_0_* variation to shift *P* gradient 3-fold in position	** $e^{B\cdot 2x}$ **
***H***	HD-ZIP III	
*prodH*	*H* production rate constant	100
*n_H_*	Hill constant	3
*K_A_*	dissociation constant ([P] at half occupancy)	17.5
*inhH*	inhibition rate constant of *K* **on** *H*	0
*k_H_*	*H* decay constant	1
*D_H_*	*H* diffusivity	1e-4
***K***	KANADI	
*prodK*	*K* production rate constant	100
*inhK*	inhibition rate constant of *H* **on** *K*	1
*k_K_*	*K* decay constant	1
*D_K_*	*K* diffusivity	1e-4
*m*	miR166 inhibition of *H*	

Values for **Figure 4A**, deviations from these for other results are noted in text.

In Eq. 1, precursor *P* is produced at rate *P_0_* = 100 within a source region from 0< *x*< *s_e_*, *P_0_* = 0 otherwise. Diffusion (*D_P_*) from the source and decay (*k_P_*) form an exponential steady-state (time-independent) gradient in *P*. For small *s_e_*, the solution of Eq. 1 is given closely (exactly, for *s_e_* = 0) by


(4), $$P=P_{0}e^{-Bx}$$

where


(5) $$B=\sqrt{k_{P}/D_{P}}$$

is the exponential decay constant, or instantaneous slope, of the *P* gradient. Increasing *B* produces spatially shorter *P* gradients (in terms of the position of a particular *P* concentration from the source region).

This SDD (source-diffusion-decay) model has been applied to a number of developmental systems, including Bcd positional information in *Drosophila* (e.g. Drocco et al., 2012). While exponential gradients may be common and could be the case for the upstream precursors of HD-ZIP III, Eq. 4 more broadly provides a means for representing the qualities of concentration level (gradient height, *P_0_* parameter) and spatial extent (gradient length, *B* parameter), which are general to any molecular concentration pre-pattern.

*H-K* positioning is considered in terms of this positional information gradient from the tip (analogous to models of anterior patterning and the Bcd gradient in *Drosophila*; Drocco et al., 2012): *H* is activated by *P* (*prodH* term, Eq. 2); *K* is activated constitutively (*prodK* term, Eq. 3) and spatially patterns in response to *H* (due to *inhK* > 0, the effect of *H* on *K*). *H* activation is represented by Hill enzyme kinetics, allowing for *P* cooperativity (*n_H_* > 1, Eq. 2).

Experiments indicate that HD-ZIP III and KAN are mutually inhibitory, with the HD-ZIP III domain advancing basipetally from the tip when KAN is decreased (Izhaki and Bowman, 2007) and retreating towards the tip when KAN is increased (Caggiano et al., 2017), and likewise for the effect of HD-ZIP III on KAN (Emery et al., 2003; Caggiano et al., 2017). This inhibition is represented *via* the *inh*_ factors in the *prod*_ terms, Eqs. 2, 3. *inhH* is the inhibition of *H* by *K*, and *inhK* is the inhibition of *K* by *H*. Biologically, HD-ZIP III - KAN inhibition can involve small RNAs. Caggiano et al. (2017) showed a role for the peripherally-expressed miR166 (in the KAN domain) in inhibiting HD-ZIP III. During later development of the leaf adaxial/abaxial boundary, tasiARF small RNAs originate from the adaxial HD-ZIP III domain (SAM side) and inhibit abaxial ARFs (auxin response factors), while miR166 from the abaxial domain inhibits HD-ZIP III (Merelo et al., 2017; Skopelitis et al., 2017). miR166 inhibition of *HD-ZIP III* expression has been demonstrated in conifer cotyledon development (Li et al., 2016). In terms of Eqs. 2 and 3, *D_H_* and *D_K_* could represent the mobility and spatial extent of small RNAs from the HD-ZIP III and KAN domains, respectively, and the *inh*_ terms could represent the inhibition strength of the small RNAs on their targets. The inhibition terms are linear in inhibitor concentrations, as indicated by the single small RNA binding site in the *HD-ZIP III* target reported in Skopelitis et al. (2017).

The effect on whorl position of the dynamics represented by each term in Eqs. 1 – 3 will be addressed in the Results. Whorl position is determined as the *x* at which the *H* and *K* patterns cross. The *H-K* trough around this crossing point (the region conducive to PIN1-auxin patterning of primordia) is determined as the region with (*H* + *K*)< 50 (*K* has a maximum concentration of 100, for the parameters in **Table 1**).

## 3 Results

### 3.1 Quantifying the increase of whorl radius with *n_c_*

**Figure 3:**
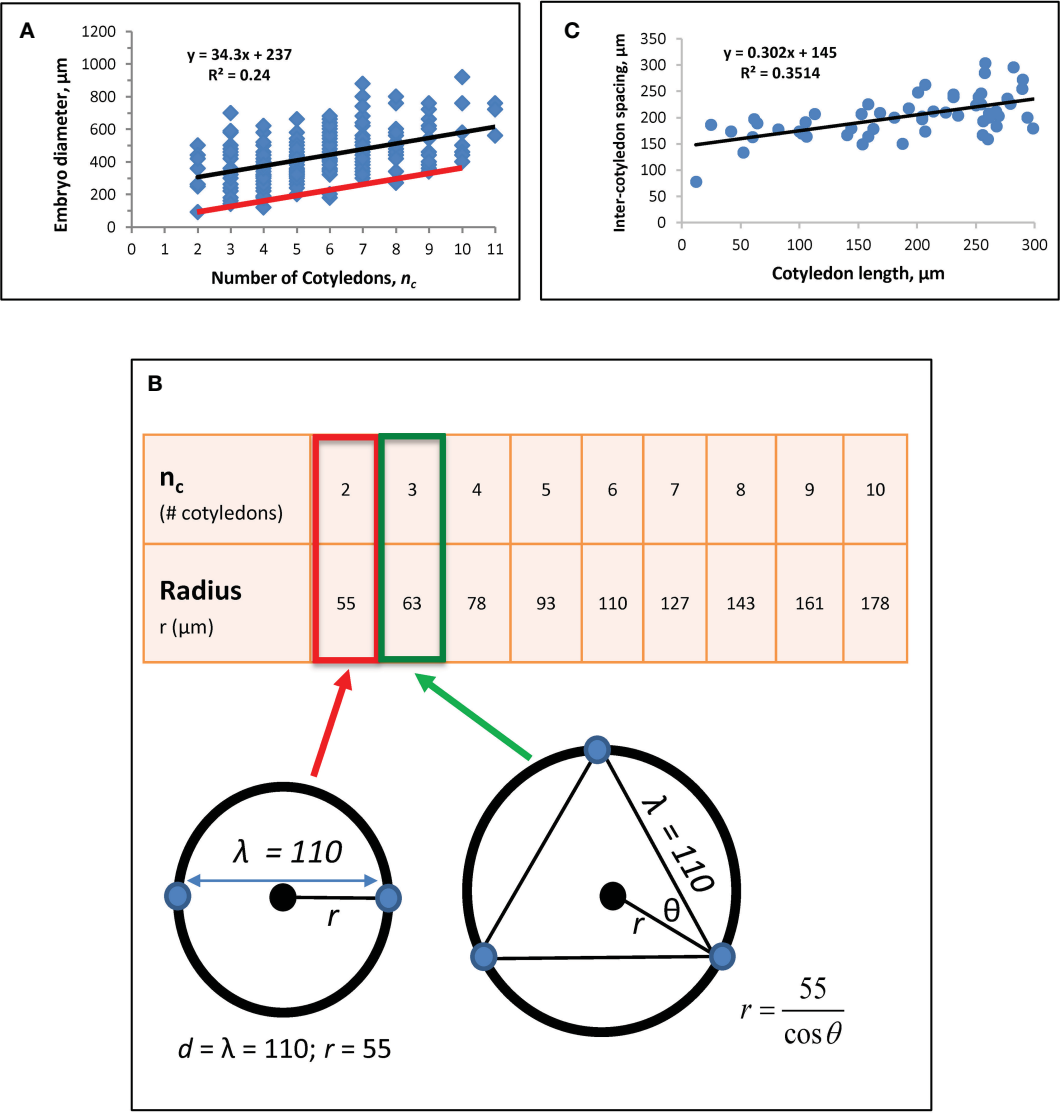
Whorl radius (*r*) corrected for growth effects. **(A)** A regression of *n_c_* vs. embryo diameter indicates λ = 110 μm (Eq. 6). Modified from Holloway et al. (2016). Black line: linear regression (equation shown) for **Figure 1A** data from *n_c_* = 2 to *n_c_* = 11. **(B)** Whorl radius, *r*, for each *n_c_*. Bottom left schematic: at *n_c_* = 2, whorl diameter equals λ, with the radius half of that, or 55 μm (red box on table). Cotyledon centres (blue dots) and whorl (black circle; corresponding to red ring in **Figure 1C**) are shown. This *r* value coincides with the smallest observed embryo diameter in the data (left end of red line in A). *r* for subsequent *n_c_* are calculated as shown on the bottom right schematic for *n_c_* = 3 (green arrow and box). Plotting these *r* values against *n_c_* on data (red line on A) coincides with the smallest observed embryos at all *n_c_*, suggesting the larger observed diameters (blue dots above red line in A) have had growth subsequent to the initial specification of cotyledon position. **(C)** Regression of directly-measured cotyledon-to-cotyledon spacing against cotyledon length (each point averaged from the multiple cotyledons in each embryo) shows an increase in spacing as cotyledons extend (and the embryo expands). The y-intercept indicates a cotyledon-to-cotyledon spacing of 145 μm at the onset of cotyledon growth.


Prior work established a correlation between *n_c_* and embryo diameter *D* (see **Figure 3A**; Harrison and von Aderkas, 2004; Holloway et al., 2016), from which an estimate for intercotyledon spacing λ was obtained using

(6) $$D=(\lambda /\pi )n_{c}+b$$

where the y-intercept *b* is the difference between embryo diameter *D* and cotyledon whorl diameter *d*: *b* = *D* - *d*. The slope of Eq. 6, *λ/π*, represents the whorl diameter change necessary to fit in each additional cotyledon on the whorl circumference. A new regression with *n* = 166 embryos (data not shown) corroborated the value of λ = 110 µm for spruce reported in Holloway et al. (2016).

To obtain an accurate estimate of the whorl radius *r* (*d*/2) set by molecular (*H, K*) patterning, the effects of tissue growth on observed diameter are factored out. For this, we note that at *n_c_* = 2, *d* = λ (**Figure 3B** schematic), giving a whorl radius *r* = 55 µm for λ = 110 µm. *r* values calculated for *n_c_* up to 10 are shown in **Figure 3B**. The approximately 3-fold radius increase from *n_c_* = 2 to *n_c_* = 10 agrees with modelling results in Holloway et al. (2018), Figure 4), which produced this *n_c_* range over a 3-fold increase in system size (*D*). Plotting *n_c_* vs. 2*r* from **Figure 3B** onto data (red line, **Figure 3A**) coincides with the smallest observed embryos, indicating that most raw measurements (data points above the red line in **Figure 3A**) include some degree of tissue expansion after primordia initiation. *b* ≈ 0 for the red line in **Figure 3A** agrees with the observation that whorl radius coincides with embryo radius (e.g., **Figures 1B, D, E**); *b* > 0 for the blue dots in **Figure 3A** indicates the degree of embryo expansion from the initial *r*. The *r*-values in **Figure 3B** (red line in **Figure 3A**) are the growth-corrected whorl radii used for matching natural whorl variation in the *H-K* patterning model (3.2).

Intercotyledon spacing λ can be estimated independently from the Eq. 6 approach by direct measurements of cotyledon-to-cotyledon spacing, together with a regression against cotyledon length (**Figure 3C**). The y-intercept of this regression represents cotyledon length = 0, or the onset of morphogenesis. To estimate the intercept, we chose *n* = 55 embryos with cotyledon length< 300 µm (where there is an early strong linear relation between embryo diameter and cotyledon length; this linear relation decreases later, as diameter expansion slows while cotyledons continue to lengthen). This gives a value of 145 µm, somewhat larger than the value of λ = 110 µm from the method in **Figure 3A**. This difference is consistent with modelling results indicating that positional specification occurs during embryo tip flattening (Nagata et al., 2013; Holloway et al., 2018); i.e. the estimate of λ = 110 µm corresponds to patterning on the earlier dome represented in **Figure 1C** (the stage shown in Holloway et al., 2016, Figure 1C), while the estimate of 145 µm would be for the later flattened dome shape preceding cotyledon extension (a stage slightly earlier than **Figure 1E**; or see Holloway et al., 2018, Figure 1D), which would be expected to have a slightly expanded radius.

We use the earlier-stage estimate of λ = 110 µm for the modelling (**Figure 3B**), but note that the relative increase of *r* with *n_c_*, specifically the over 3-fold increase in *r* from *n_c_* = 2 to *n_c_* = 10, depends on the angle between cotyledons (θ, **Figure 3B**), not on the absolute value of the intercotyledon spacing.

### 3.2 Positional control of the whorl radius (distance from the apex or tip)

**Figure 4:**
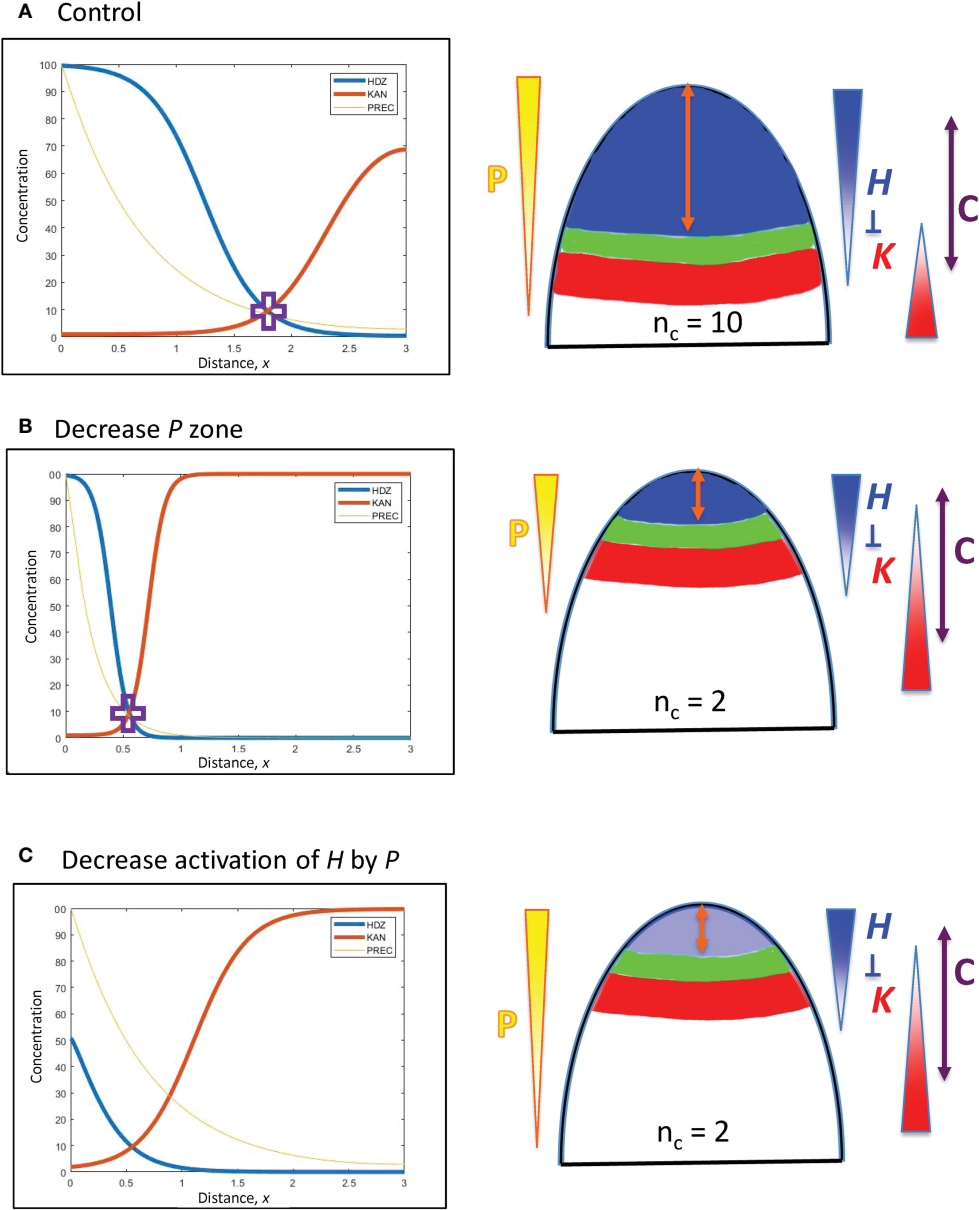
Upstream (*P*) and *H* response factors can affect the positioning of the HD-ZIP III (*H*) domain. Left: Concentration vs. distance from the tip *x* for the three model variables (Eqs. 1 – 3): *P* (Precursor or meristematic factors, yellow); *H* (HD-ZIP III, blue); and *K* (KAN, red). Right: Diagrams of (i) the expression domains of *H* (blue) and *K* (red) on the embryo (or SAM), (ii) the gap, or trough, between them (green) in which lateral organs can be initiated, and (iii) the orange double arrow indicating the distance of this gap from the tip. The expected *n_c_* for these distances is shown, and *P*, *H*, *K* gradients are indicated, as well as *H-K* inhibition interactions. The double-headed purple arrow, C, indicates the region in which cells are competent to form primordia, if *H* and *K* concentrations are low enough. In this figure, *H*-*K* inhibition is one-way (not mutual): inhibition of *K* by *H* (*inhK* = 1) patterns *K* in response to the *H* pattern, but there is no inhibition or effect on *H* by *K* (*inhH* = 0). **(A)** Starting position, the *H*-*K* crossing point (purple cross) is at a distance *x* = 1.8 (= 180 µm) from the tip, corresponding to the whorl radius for *n_c_* = 10 (**Figure 3B**; slightly lower *n_c_* on a curved geometry, see section 2). **(B)** Changing the extent of the *P* zone: increasing the decay constant *B* (Eq. 5) by a factor of 10/3 (=1.8/0.54) shifts the position of the *P* concentration corresponding to the *H*-*K* crossing point (purple cross) by the same factor, to *x* = 0.54 (corresponding to *n_c_* = 2), illustrating the linear *B*-shift shown in Eq. 8. The steeper *P* in **(B)** induces a steeper *H* slope (shorter *H* domain) than in **(A)**. **(C)** Changing the *H* activation strength: the dissociation constant *K_A_* sets the *P* concentration at which *H* will be half-maximal. *P* varies 5.6-fold from *x* = 1.8 to *x* = 0.55. Increasing *K_A_* (decreasing the activation of *H* by *P*) by the same factor (5.6) shifts the *H-K* interface position from x = 1.8 **(A)** to *x* = 0.55, resulting in *n_c_* = 2 **(C)**.

The model in Eqs. 1 – 3 represents a general approach for characterizing control of the position from the tip of the *H-K* interface at which cotyledons or leaves form, i.e. for conifers, control of the whorl radius *r*. Perturbations are done from a parameter set that produces an *H-K* pattern corresponding to an observed *r* (e.g. 180 µm, **Table 1** and **Figure 4A**). Patterning and shifting results are not affected by balanced changes in parameters. For example, doubling both *prodH* and *prodK* values (changing absolute concentrations) does not change the results presented in 3.2.1 and 3.2.2.1, but altering *prodK* alone (i.e. changing it relative to *prodH*) produces a positional shift (3.2.2.2). The results below characterize the relative strengths of positional shifts induced by perturbations in each aspect of the model. These can occur at three general levels: a) in the patterning upstream of *H* (i.e. Eq. 1; meristem factors); b) in the response of *H* to upstream pattern (Hill term in Eq. 2); and c) in the mutual inhibition of *H* and *K* (*inh*_ terms in Eqs. 2, 3). For the natural 3-fold variability in *r* observed in conifer embryos (**Figure 3B**), variation at a), b), or c) could suffice. We characterize the parameter variation in each of these levels to drive 3-fold positional change (3.2.1.1, 3.2.1.2, 3.2.2.1, respectively). Positional shifts induced by experimental perturbation of *H* or *K*, by contrast, indicate a key role for *H*-*K* mutual inhibition in positioning (c), discussed in section 3.2.2.2.

#### 3.2.1 Spatial extent of the HD-ZIP III (*H*) apical domain

##### 3.2.1.1 Upstream (precursor, e.g. meristem) patterning (*P* effects) on whorl position

In Eq. 4 for the concentration gradient of *P*, perturbations to *P_0_*, *B*, or *s_e_* shift the position *x* at which a particular concentration *P* occurs. (In **Figure 2**, *P_0_* is the maximum height of the red curve *P*; *s_e_* is the spatial extent of the flat part of *P*; *B* is the curvature of the curved part of *P*.) This affects where the downstream *H* will be activated, and hence the position of the *H-K* interface and the corresponding whorl radius *r*.

***s_e_* perturbations:** Increasing *s_e_* (the spatial extent of the *P* source) shifts *P* basipetally (away from the tip), with *x*-increase proportional to *s_e_* increase. Such variation in *s_e_* would reflect upstream patterning regulation (e.g. meristem size establishment), not regulation at the *H*, *K* level.

***P_0_* perturbations:** represent dosage differences, with respect to how much upstream regulator is produced, for example, in each embryo during cotyledon formation. Say a particular *P* concentration is associated with a position from the tip of *x* = 55 µm (corresponding to the *r* for *n_c_* = 2; **Figure 3B**), given by $P=P_{0}e^{-Bx}$ . The factor of *P_0_* required to move this *P* three-fold in distance to 3*x* = 165 µm, $P=aP_{0}e^{-B\cdot 3x}$ , is given by:


(7), $$a=e^{B\cdot 2x}$$

which depends exponentially on the decay length *B* of the gradient. (See notes regarding surface curvature in the section 2: a surface distance of 165 µm corresponds to *r* = 165 µm on a flat surface, but would correspond to *r* = 139 µm on a hemisphere of radius 165 µm. These geometric effects do not have a large effect on *n_c_*, the two *r*-values correspond to *n_c_* = 8 or 9, **Figure 3B**.) For the *a*-factor to be linear with *x* (i.e. *a* = 3 for 3*x* = 165 µm, or 1.65 computational spatial units), Eq. 7 shows that B=ln31.1=1 (per computational spatial unit). Well-defined localized upstream patterns (e.g. meristem regions) have steeper *P* gradients than this, with *B* > 1. In such cases, *a*-factor (i.e. dosage) variation would have to be larger than 3-fold to drive a 3-fold shift in *x* position. For instance, the *P* gradient in **Figure 4A** (*B* = $\sqrt{2}$ ≈ 1.41) would require a 5.46-fold drop in *P_0_*, from 100 (shown) to 18.3, to shift *x* 3-fold from 1.8 (shown) to 0.6; the *P* gradient in **Figure 4B** (*B* = 4.71) would take a 178-fold increase in *P_0_*, from 100 (shown) to ~17,800, to shift *x* 3-fold from 0.55 (shown) to 1.65.

***B*, perturbations in the decay length of the gradient:** Pushing a *P* associated with *x* = 55 µm (corresponding to *n_c_* = 2), $P=P_{0}e^{-B_{1}x}$ , to a position 3-fold larger (165 µm, corresponding to *n_c_* = 8 or 9) by changing *B*, $P=P_{0}e^{-B_{2}\cdot 3x}$ , requires that


(8) $$B_{1}=3\cdot B_{2}$$

i.e., the position from the tip *x* depends linearly on decay length *B*. Such a shift is illustrated in **Figures 4A, B**. *B* has a square root dependence on its underlying physical parameters (Eq. 5): a 9-fold change in the (rate constant *k_P_* : diffusivity *D_P_*) ratio would be needed for a 3-fold change in *x*.

##### 3.2.1.2 Response of *H*

For a given upstream *P* gradient and no inhibition on *H* by *K* (*inhH* = 0), the steepness of the *H* concentration profile and the spatial extent of its expression domain are determined by the activation kinetics (1^st^ bracket, Eq. 2).

**Figure 5:**
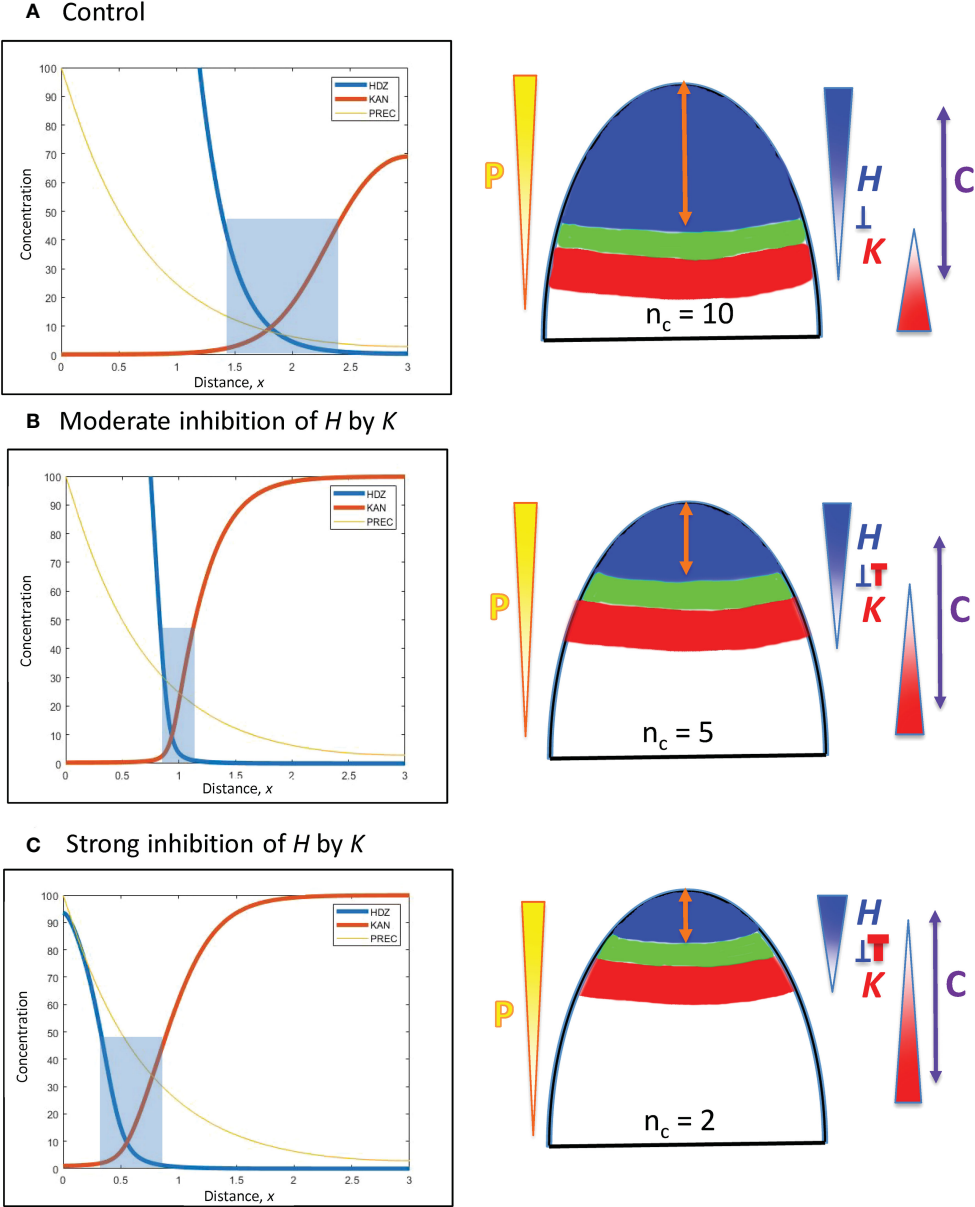
*H-K* mutual inhibition can produce the natural 3-fold variation in whorl position due to increasing *K* inhibition on *H*. Left: Concentration vs. distance from the tip *x* for the three model variables, as in **Figure 4**. Right: Diagrams of the domains on the embryo or SAM, as in **Figure 4**. **(A)** No mutual inhibition; *inhH* = 0; crossing point occurs at *x* = 1.8, the same reference position as **Figure 4A**. **(B)** *inhH* = 2.4; crossing point occurs at *x* = 0.95. **(C)** *inhH* = 4.9; crossing point occurs at *x* = 0.55. For positional shifts induced in this way, smaller *n_c_* would be associated with stronger inhibition of *H*. by *K*. Blue rectangles indicate the trough zone where (*H*+*K*)< 50, permissive for primordia initiation. In **Figures 5**–**8**, baseline *prodH =* 600, *K_A_* = 32.

**Figure 6:**
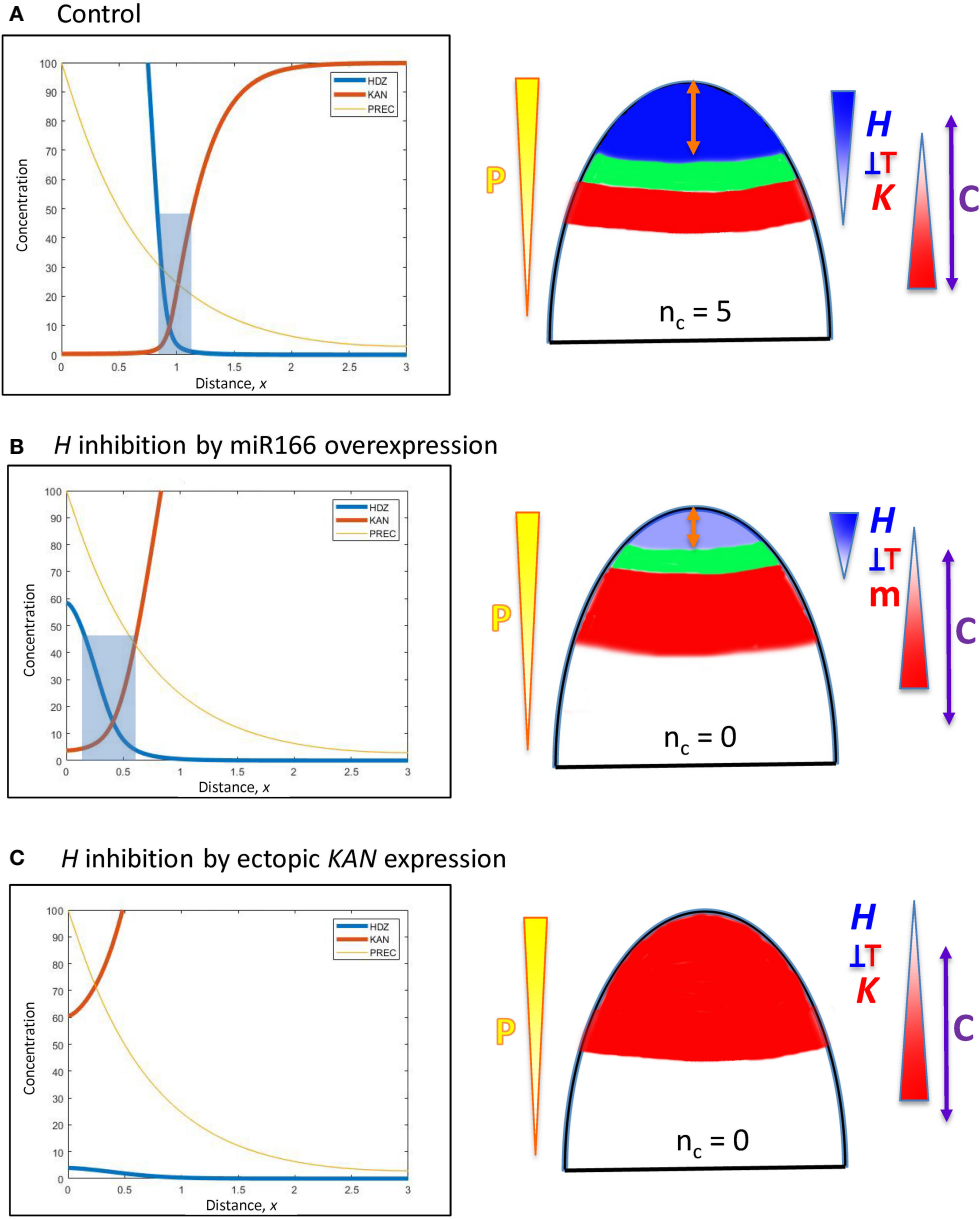
Modelling experimentally-induced acropetal shifts in the *H-K* interface due to increased inhibition of *H*. Diagrams and colouring are as in **Figure 5**. **(A)** All computations start from the *H-K* interface position of *x* = 0.95 (same as **Figure 5B**, corresponding to the commonly observed *n_c_* = 5, **Figure 1A**). **(B)** Experiment: *miR166* overexpression in larch reduces *HD-ZIP III* expression and reduces the prevalence of cotyledon forming embryos (Li et al., 2016). Model: increasing *prodK* (assumed proxy for miR166, m) decreases *H*_max_ and shifts the *H-K* interface acropetally to a region too small to form cotyledons. **(C)** Experiment: ectopic *KAN* expression in the SAM results in no leaves in *Arabidopsis* (Caggiano et al., 2017). Model: even higher *prodK* (than B) eliminates the *H-K* interface and the permissive zone for primordia initiation. This would correspond to *n_c_* = 0 in conifers.

**Figure 7:**
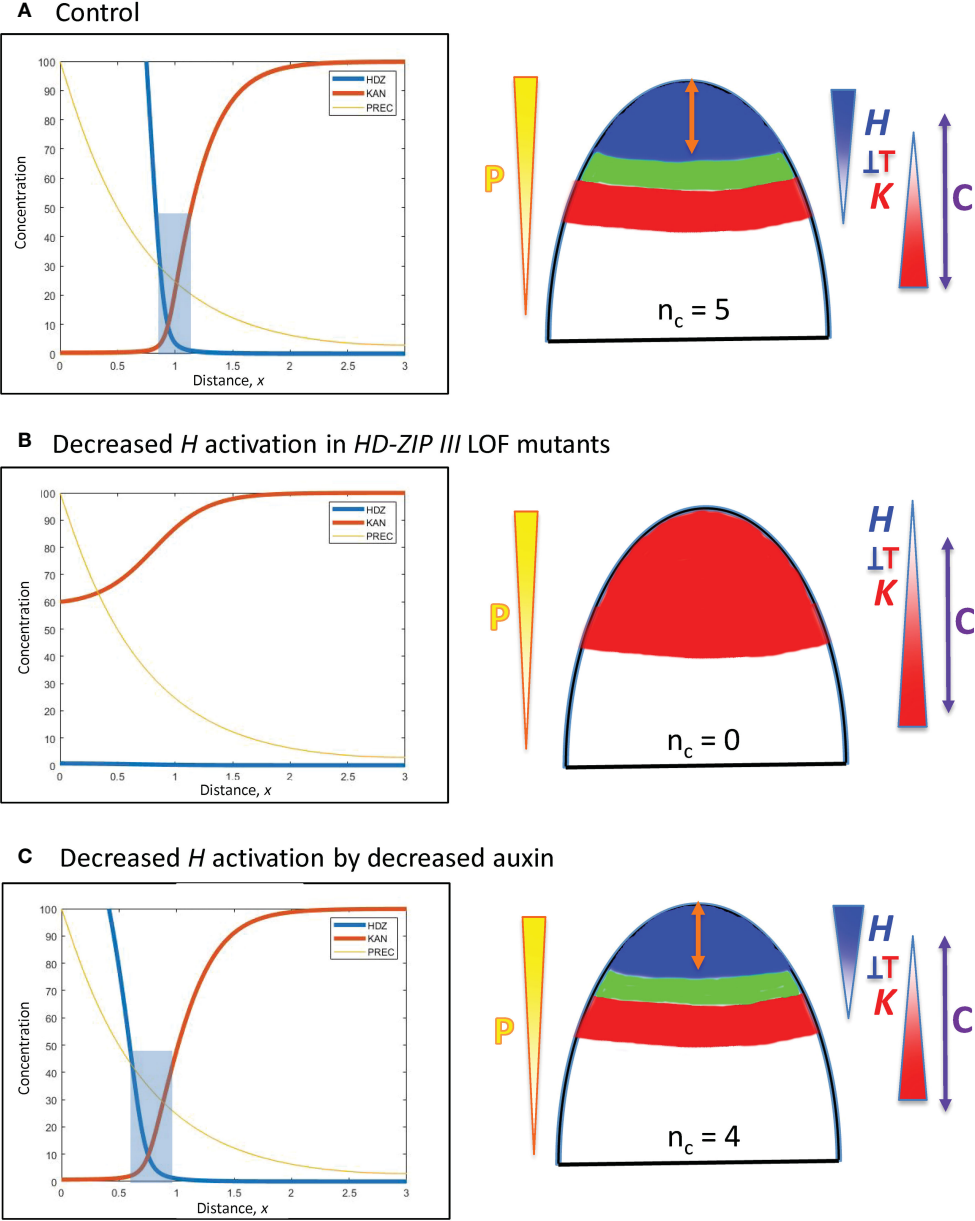
Modelling experimentally-induced acropetal shifts in the *H-K* interface due to decreased activation of *H*. Left, right and colouring as in **Figure 5**. **(A)** All computations start from an *H-K* interface position of *x* = 0.95 (**Figure 5B**). **(B)** Experiment: *HD-ZIP III* loss-of-function mutants (e.g., *phb-6 phv-5 rev-9* triple mutant) in *Arabidopsis* have reduced cotyledon and leaf formation (Emery et al., 2003; Prigge et al., 2005; Huang et al., 2014). Model: a 6-fold *prodH* decrease eliminates the *H-K* interface, resulting in no cotyledon production. **(C)** Experiment: auxin decrease in the *Arabidopsis* SAM shifts the *H-K* interface acropetally (Caggiano et al., 2017). Model: 3-fold *prodH* decrease shifts the *H-K* interface the observed 20 µm, to *x* = 0.76.

**Figure 8:**
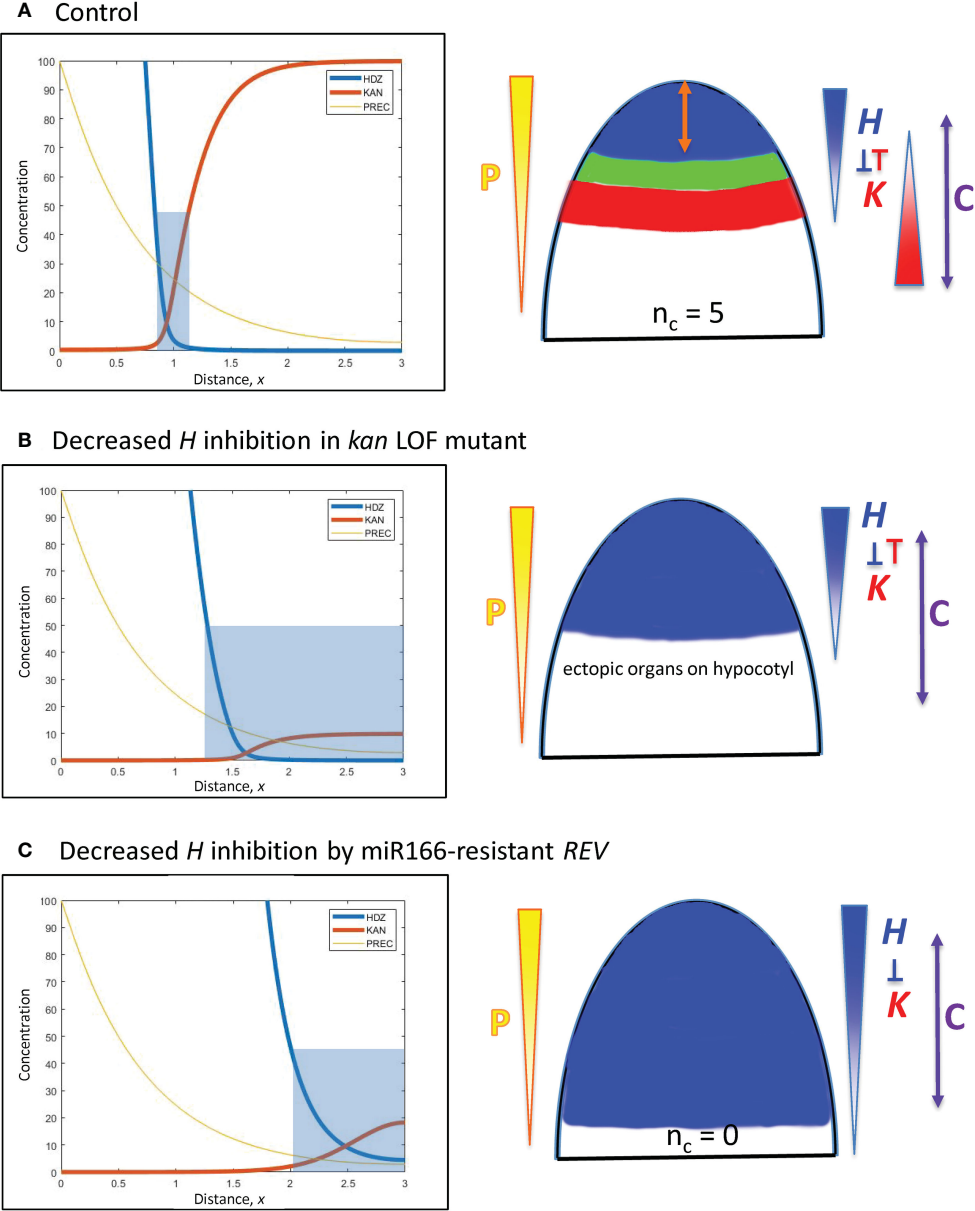
Modelling experimentally-induced basipetal shifts in the *H-K* interface due to decreased inhibition of *H.* Diagrams and colouring are as in **Figure 5**. **(A)** All computations start from *x* = 0.95 (**Figure 5B**). **(B)** Experiment: *kan1 kan2 kan4* triple loss-of-function mutant in *Arabidopsis* produces ectopic leaves on the hypocotyl (Izhaki and Bowman, 2007). Model: a strong decrease in *prodK* removes basal inhibition and results in ectopic basal organs. **(C)** Experiment: a miR166-resistant *rev* mutant in *Arabidopsis* produces no leaves (Caggiano et al., 2017). Model: *inhH* = 0, plus strong increase in *prodH* (ectopic overexpression) flattens *K* and pushes *H* far from the tip to a region where cells may not be competent to initiate lateral organs (beyond the C arrow).

**Steepness of the *H* domain:** Hill kinetics can represent the contribution of nonlinear, or cooperative, activation for *n_H_* > 1. Linear or weakly nonlinear activation (*n_H_* = 1, 2) produces low-slope *H* gradients, characterized by smaller concentration differences over a given distance than higher-slope patterns. Lower-slope gradients provide poorer discrimination between inhibited and uninhibited (primordia initiation) regions according to *H* concentration, and are more susceptible to shifting or even loss of an *H-K* interface for concentration variations in say *P* or *H* (any given concentration change translates into a larger positional shift on a shallower concentration gradient than on a steeper one). Images in *Arabidopsis* indicate that HD-ZIP III and KAN are in sharp well-defined domains (Caggiano et al., 2017), consistent with *n_H_* ≥ 3. The *n_H_* = 3 used in **Figures 4**–**8** produces a high-slope *H* concentration gradient while keeping the *H-K* trough relatively wide. Steeper slopes (*n* > 3) decrease trough widths (**Table 2**, 3.2.2.1), limiting the width of the uninhibited zone for primordia initiation.

**Table 2:** *H* domain steepness affects trough width and shiftability.

*n_H_* ^1^	*inhH* change needed to shift *H-K* interface from *x* = 1.8 to *x* = 0.55	trough width for *H-K* interface at *x* = 0.95^2^
1	6.7e-2	0 (*H*+*K* above threshold)
2	6.2e-1	0.33
3	4.9 (shown in **Figures 5A, C**)	0.29 (shown in **Figure 5B**)
4	2.2e1	0.25
5	1.5e2	0.20
6	1.2e3	0.16

^1^Increasing *n_H_* increases *H* slope.

^2^Trough positions are defined by (*H+K*)< 50.

**The spatial extent of the *H* domain:** depends on the dissociation constant *K_A_* in Eq. 2, representing the concentration of *P* needed to induce a given *H* response (specifically, *K_A_* is the *P* concentration producing half-maximal *H*). *K_A_* shifts produce *x*-shifts in direct proportion to the change in *P* between positions. For instance, for the *P*-gradient in **Figure 4A**, *P* changes by a factor of 5.6 between *x* = 1.8 and *x* = 0.55. A 5.6-fold increase in *K_A_* shifts the *H*-*K* boundary from *x* = 1.8 (**Figure 4A**) to *x* = 0.55 (**Figure 4C**). The relation of the *P* values to the x-shifts, for instance $P_{1}/P_{2}=e^{B\cdot 2x}$ for a 3*x* shift, has the same exponential dependence on *B* as for the *P_0_* dosage response (Eq. 7). That is, a given *x*-shift can be induced by a smaller *K_A_* change on a flatter low-*B* gradient than on a steeper high-*B* gradient. Variation in the response to upstream pattern (how strongly *P* activates *H*) could underlie the 3-fold positional variation observed for conifer cotyledon whorls, but, like dosage responses (3.2.1.1), would depend on the *P* exponential decay constant (*B*).

#### 3.2.2 The effect of mutual inhibition on the *H-K* interface position

The effects of perturbing HD-ZIP III or KAN directly indicate a role for *H-K* mutual inhibition (*inhH* and *inhK* both greater than zero) in positioning the *H-K* interface (Li et al., 2016; Caggiano et al., 2017). 3.2.2.1 addresses *H*-*K* mutual inhibition in generating 3-fold positional shifts for conifer cotyledons; 3.2.2.2 addresses the dynamics corresponding to the observed positional shifts in experimental HD-ZIP III or KAN perturbations, in conifers or *Arabidopsis*.

##### 3.2.2.1 *H-K* interactions and the natural 3-fold variability in whorl radius

*H*-*K* interactions are represented by the inhibition terms in Eqs. 2 and 3. These are a product of the *inh_* inhibition strengths and the *H*, *K* concentrations. These latter can be altered by the *prod_* terms, but the effect on positional shifting is modulated through the spatial expression patterns of *H* and *K*. Variation in the *inh*_ terms provides a more direct shift of the *H-K* interface position. This is demonstrated with *inhH* variation (the inhibition of *H* by *K*) in **Figure 5**.

From a starting interface position of *x* = 1.8 (as in **Figure 4A**) with no mutual inhibition (*inhH* = 0; **Figure 5A**), increasing *inhH* pushes the interface acropetally (to smaller *x* distance from the tip). For *n_H_* = 3, *inhH* = 2.4 shifts the interface to *x* = 0.95 (**Figure 5B**), the position corresponding to the commonly observed *n_c_* = 5 (see **Figures 1A**, **3B**); *inhH* = 4.9 shifts the interface to *x* = 0.55 (corresponding to *n_c_* = 2; **Figure 5C**). Variation in *inhH* (potentially variation in the miR166 inhibition strength on HD-ZIP III) could be a means for producing whorl radius and therefore cotyledon variability. Typical *n_c_* (such as 5) would have partial *H* inhibition by *K*, lower *n_c_* would have stronger inhibition than this, and higher *n_c_* would have weaker inhibition.

Variation in *inhK* (the inhibition of *K* by *H*) can behave similarly to *inhH*: a 2-fold shift from *x* = 0.55 (**Figure 5C**) to *x* = 1.1 can be induced by a roughly 3.5-fold decrease in *inhH* or increase in *inhK*.

Positional shifting is affected by the steepness of the *H-K* interface. **Table 2** shows the increasingly large *inhH* changes needed to drive the *H-K* interface from *x* = 1.8 to *x* = 0.55 as the slope (controlled by *n_H_*) increases. Steeper *H-K* interfaces have relatively lower concentrations of *K* under the *H* domain, requiring higher *inhH* for equal effect on *H* (see inhibitory term, Eq. 2), and hence require higher *inhH* for the same positional shift.

As mentioned in 3.2.1.2, the width of the *H-K* trough (the region permissive for PIN1-auxin patterning of primordia) is also affected by gradient steepness. **Table 2** shows the narrowing of troughs for increasing *n_H_* (steepness) at *x* = 0.95 (corresponding to *n_c_* = 5). (Images in *Arabidopsis* (Caggiano et al., 2017, Figure 1) indicate trough widths of 10 – 20 µm; cotyledon diameters of 110 µm could indicate wider *H-K* troughs in conifer embryos.)

Similar shifting and trough width effects are seen when changing the steepness of the *H* expression profile through other means, such as upstream changes in *P* (see **Figures 4A, B**) or changes in transport rates. For example, increasing *D_H_* or *D_K_* in Eqs. 2, 3 lowers the *H* and *K* gradients and broadens troughs, but also affects the *prodH* needed in the model. Variation in small RNA inhibitor diffusion (e.g. of miR166) could potentially affect trough width independently of *H* production.

The results indicate that a moderate steepness *H-K* interface (represented by *n_H_* = 3) can respond to moderate *inhH* variation with the 3-fold positional shifting and *n_c_* variability observed in conifer cotyledon development: too sharp (such as *n_H_* > 3) decreases shiftability; too shallow (e.g. *n_H_* < 3) produces poorly defined troughs (lacking the distinct switching from high to low HD-ZIP III and KAN levels observed in *Arabidopsis*; Caggiano et al., 2017).

##### 3.2.2.2 Experimental perturbations of HD-ZIP III and KAN

Several experiments (one in larch, six in *Arabidopsis*) directly implicate HD-ZIP III - KAN mutual inhibition in setting their interface position. These can be classified as acropetal shifts (moving the interface towards the tip, or smaller distance *x* from the tip) or basipetal shifts (moving the interface away from the tip, towards larger distance *x* from the tip). All simulations below (**Figures 6**–**8**) start from *x* = 0.95 (**Figure 6A**, the same reference pattern as **Figure 5B**), corresponding to the commonly observed *n_c_* = 5 (**Figure 1A**).

**Acropetal shifts in the *H-K* interface towards the tip**

*Increased inhibition of H at the tip*

A. In larch embryos, *miR166* inhibitor overexpression by 2- to 4-fold reduces *HD-ZIP III* expression and decreases the prevalence of cotyledon formation (Li et al., 2016). The inhibition of *H* by *K* in Eqn. 2 could represent a direct interaction of KAN or an indirect interaction *via* miR166 (miR166 is associated with the KAN domain in *Arabidopsis*). Representing *miR166* overexpression as an increase in *K*, a 2.2-fold *prodK* increase produces a 5-fold decrease in *H*_max_ and shifts the interface position to *x* = 0.41 (**Figure 6B**). While less *miR166* overexpression could give smaller positional shifts than this and allow cotyledon formation (with *n_c_* lower than normal), shifts to *x*< 0.55 (such as shown in **Figure 6B**) would not have sufficient space (at λ ≈ 110 µm) to form multiple cotyledons in a ring, resulting in ring or pin-formed phenotypes (*n_c_* = 0 or 1).
B. In the *Arabidopsis* SAM, ectopic *KAN* expression eliminates leaf production (Caggiano et al., 2017). This is represented in the model by increasing *prodK.* 3-fold increase in *prodK* flattens *H* expression and eliminates the *H-K* interface or trough (**Figure 6C**). In this case, inhibition of PIN1-auxin patterning (from *K*) would extend over all positions, with no permissive zone for primordia initiation.
*Decreased activation of H at the tip*
C. In a complementary fashion, *HD-ZIP III* loss-of-function mutations in *Arabidopsis* reduce cotyledon and leaf formation. In particular, double or triple mutants can have 0, 1 or 2 cotyledons compared to the wild-type *n_c_* = 2 (Prigge et al., 2005, Figure 2; Huang et al., 2014, Figure 6), as well as lacking leaf formation (Emery et al., 2003, Figure 5). Loss-of-function is modelled by an overall drop in *H* production. A strong (6-fold) reduction in *prodH* eliminates the *H-K* interface and the auxin-permissive zone for primordia initiation (**Figure 7B**), resulting in *n_c_* = 0; a smaller reduction in *prodH* shifts the *H-K* interface acropetally without eliminating it (data not shown), giving a reduced but non-zero *n_c_*.
D. Auxin decrease shifts the REV*-*KAN interface acropetally by about 20 µm on the *Arabidopsis* SAM (Caggiano et al., 2017), suggesting auxin activation of *H* tip expression. Reduction of this activation is modelled by a 3-fold reduction in *prodH*: this shifts the *H-K* interface from *x* = 0.95 to *x* = 0.76 while maintaining the *H-K* interface and trough (**Figure 7C**). Auxin activation could also be represented *via P*, in which case auxin reduction would correspond to *P_0_* reduction (Eq. 1), shifting position as indicated by Eq. 7. In conifer whorls, at typical *n_c_* values, a 20 µm shift would correspond to a change in one cotyledon, e.g. from *n_c_* = 5 to *n_c_* = 4 (**Figure 3B**).

**Basipetal shifts in the *H-K* interface away from the tip**

E. *Decreased inhibition of H from the base*: *kan1 kan2 kan4* loss-of-function mutants in *Arabidopsis* produce ectopic leaves on the hypocotyl (Izhaki and Bowman, 2007). Simulating loss-of-function by a 10-fold reduction in *prodK* removes the outer boundary of the *H-K* interface, creating a large basal zone permissive for primordia initiation (**Figure 8B**).
F. *Decreased inhibition of H at the tip*: Ectopic overexpression of miR166-resistant *REV* in *Arabidopsis* expands REV basipetally and results in no leaves (Caggiano et al., 2017). Loss of inhibition is simulated by setting *inhH* = 0 (miR166 resistance); ectopic overexpression is simulated by a 100-fold increase in *prodH*. This flattens *K* and pushes *H* basipetally (**Figure 8C**). If *H* is pushed far from the tip, its boundary could potentially extend beyond tissue that is competent to form primordia. For example, *H* > 50 inhibits primordia within 200 µm of the tip in **Figure 8C**; if tissue beyond 200 µm was not competent, no leaves would form.
G. *Increased activation of H at the tip*: Gain-of-function mutations in *HD-ZIP III* can result in *n_c_* = 3 in *Arabidopsis*, compared to the wild-type *n_c_* = 2 (Huang et al., 2014, Figure 6). Over-expression was modelled as an increase in *prodH*. 2-fold increase in *prodH* (from **Figure 5B** conditions) resulted in a basipetal shift of the *H-K* boundary from 0.95 to 1.15 (data not shown), with an intact *H-K* interface trough. This 20 µm shift would correspond to an increase in *n_c_* by one using conifer values (**Figure 3B**).

## 4 Discussion

Cotyledons and true leaves are initiated in peripheral zones around embryo tips or SAMs, respectively (**Figure 2**). This initiation zone is molecularly identified as an interface between the apical domain of HD-ZIP III and the basal domain of KAN (Emery et al., 2003; Prigge et al., 2005; Huang et al., 2014). From their results, Caggiano et al. (2017) proposed that HD-ZIP III and KAN both inhibit auxin, leaving a maximum of auxin activity in the HD-ZIP III – KAN interface zone. This is consistent with the role of PIN1-auxin PAT in regulating primordia development within the initiation zone (Reinhardt et al., 2003; Hakman et al., 2009), and auxin’s role in morphogenetic outgrowth (Heisler, 2021).

Regulation of the size of the HD-ZIP III – KAN interface zone controlling auxin activity, particularly its distance from the tip, is likely to be a major determinant of cotyledon or leaf phyllotaxy. For example, Jönsson et al. (2006) switched phyllotaxy in a leaf initiation simulation by increasing the radius of the initiation zone; and molecular perturbations expected to move the HD-ZIP III – KAN interface zone acropetally or basipetally respectively reduced or increased *n_c_* in *Arabidopsis* (Prigge et al., 2005; Huang et al., 2014).

Cotyledon patterning in conifer embryos offers a unique system for exploring HD-ZIP III – KAN interface positioning, to account for the wide range of whorl radii associated with naturally variable *n_c_*. Whorl radii vary over a range of ~ 100 µm from low to high *n_c_* (**Figure 3B**), similar to variation in the gap gene *hunchback* (*hb*) expression boundary (separating anterior from posterior fates) between small and large *Drosophila* embryos (Cheung et al., 2014). *hb* boundary positioning is influenced by other gap genes, as well as a morphogen gradient of the upstream factor Bicoid (Bcd). HD-ZIP III – KAN interface positioning, similarly, is influenced by mutual interaction and upstream meristematic factors. Commonalities in these mechanisms may offer insight into size regulation in animals and plants. That is, size regulation of the auxin-active primordia initiation zone influencing phyllotaxy in plants may share features with the actively studied issue of Bcd-*hb* regulation of the mid-embryo position in variable-sized *Drosophila* embryos.

**Figure 9:**
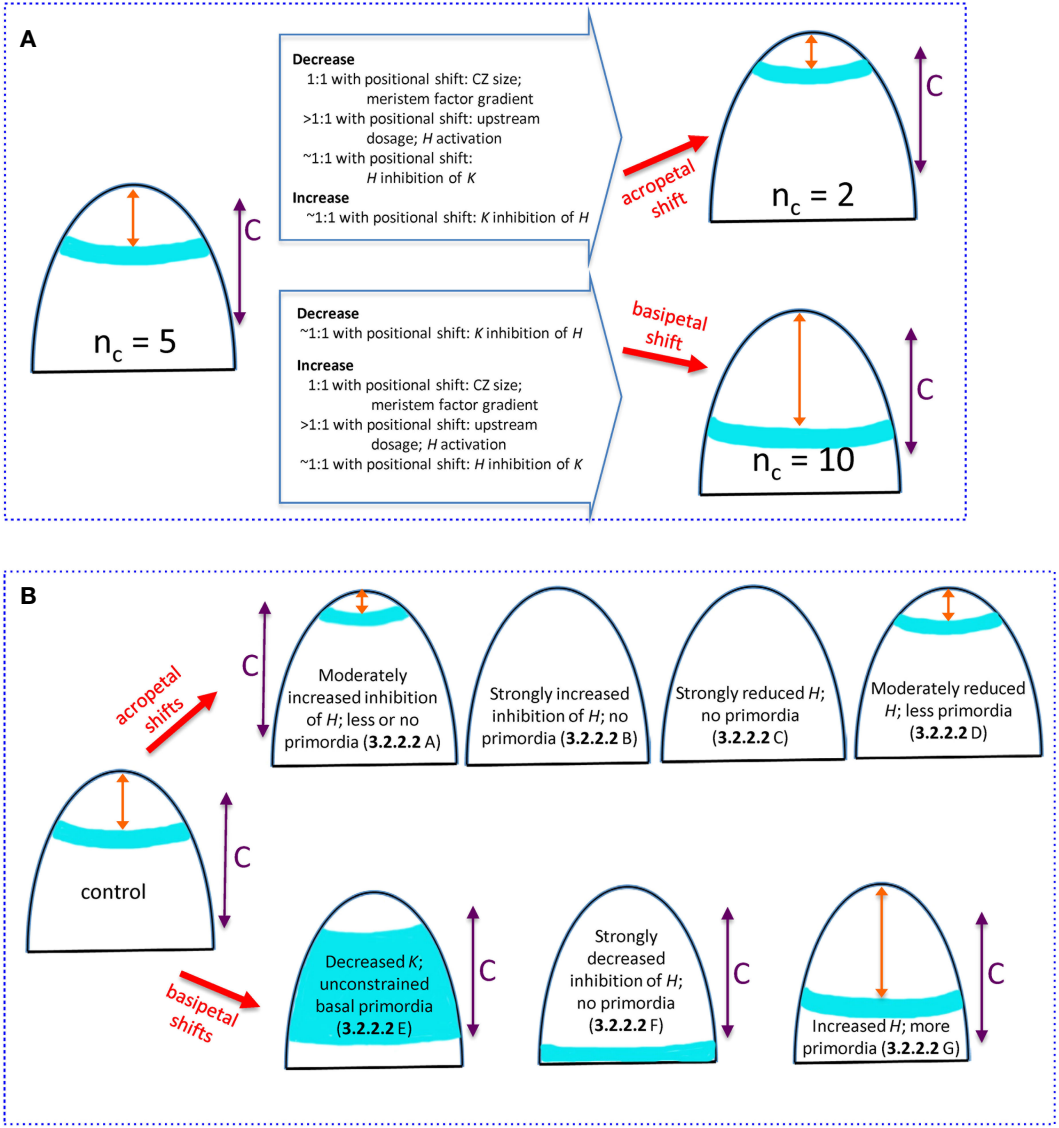
Summary of how changing *H-K* mechanism parameters can alter the whorl position and *n_c_*. All images show (i) whorl position (blue), (ii) distance *x* from the tip to the whorl (orange double arrow), (iii) competent zone (C, purple double arrow) where tissue can form primordia within the high auxin region. **(A)** Natural 3-fold range in conifer cotyledon whorl position due to variation in upstream (3.2.1.1), *H*-response (3.2.1.2), and *H-K* inhibition factors (3.2.2.1). Starting from the commonly-observed *n_c_* = 5, acropetal shifts of the whorl position reduce *n_c_* (upper) while basipetal shifts increase *n_c_* (lower). Changes in mechanism parameters, with ratio to induced positional shifts, are summarized on the large arrows. Model parameters: CZ size, *s_e_*; meristem factor gradient, *B*; upstream dosage, *P_0_*; *H* activation, *K_A_*; *H* inhibition of *K*, *inhK*; *K* inhibition of *H*, *inhH*. **(B)** Experimentally induced shifts of the primordia initiation zone due to *H-K* interactions (3.2.2.2). Acropetal shifts (upper images): A- overexpressed *miR166* (Li et al., 2016) modelled as increased *prodK*, which decreases whorl position *x* sometimes less than the space needed for multiple cotyledons; B- *KAN* overexpression (Caggiano et al., 2017) modelled as strongly increased *prodK* that eliminates the auxin-high initiation zone; C- *HD-ZIP III* loss-of-function (Emery et al., 2003; Prigge et al., 2005; Huang et al., 2014) modelled as strongly decreased *prodH* also eliminates the auxin-high initiation zone; D- auxin decrease (Caggiano et al., 2017) modelled as moderately decreased *prodH* shifts the ring acropetally. Basipetal shifts (lower images): E- *KAN* loss-of-function (Izhaki and Bowman, 2007) modelled as decreased *prodK* removes the basal border of the high auxin zone, and primordia can form in all competent tissue basal of *H* expression; F- inhibition resistant *HD-ZIP III* (Caggiano et al., 2017) modelled as zeroed *inhH* and increased *prodH* pushes the auxin-high zone basal of tissue competent to form primordia; G- *HD-ZIP III* gain-of-function (Huang et al., 2014) modelled as increased *prodH* pushes the auxin-high zone more basally, increasing the whorl radius and *n_c_*.

Work here has quantified conifer cotyledon whorl radius variation (3.1), and characterized the degree to which components of an *H-K* interface positioning mechanism could account for this variation in whorl size and the correlated *n_c_* (3.2.1, 3.2.2.1). These results on natural variability are extended to aspects of the *H-K* positioning mechanism that could account for experiments shifting HD-ZIP III and KAN localization acropetally or basipetally (3.2.2.2). The factors for natural variability in whorl radius and *n_c_* are summarized in **Figure 9A**; factors for HD-ZIP III and KAN experimental perturbations are summarized in **Figure 9B**.

*Influence of upstream (P) and response (H) factors on n_c_*

For *n_c_* variability in conifers, we can examine the degree to which specific mechanism components need to be perturbed to generate the observed 3-fold variation in whorl radius (summarized in the open arrows on **Figure 9A**). For upstream activators of *H* (e.g. meristem factors, represented by *P*, Eq. 1), variation in the core area of upstream expression (*s_e_* parameter variation; CZ, **Figure 2**) would be expected to shift the *H-K* interface (red dashed line, **Figure 2**) in direct proportion (one-to-one). Similar 1:1 shifts would occur with changes in the upstream expression profile steepness (curvature of red curve in **Figure 2**; *B* parameter, Eq. 8, corresponding to quadratic changes in underlying physical parameters, Eq. 5). That is, 3-fold changes in whorl position could be driven by 3-fold changes in upstream pattern spatial extent or steepness.

In contrast, overall concentration, or dosage, variation in the upstream factors generally needs to be relatively greater than the positional shift, particularly for upstream domains with well-defined (sharp) boundaries (large *B*), as is observed for HD-ZIP III and KAN in *Arabidopsis* (Caggiano et al., 2017). In these cases for instance, a 3-fold positional shift would require a greater than 3-fold dosage change. In general, given shifts in the *H-K* interface position (and whorl radius and *n_c_*) could be induced by smaller changes in upstream extent (CZ size) or gradient steepness than in upstream dosage. *H* activation, represented by the *K_A_* dissociation constant (Eq. 2), influences where on the upstream (*P*) gradient *H* will be expressed. The positional effect of varying *H* activation depends on the steepness of the upstream *P* gradient (red curve, **Figure 2**): steeper upstream gradients require larger changes in *H* activation than do shallower ones. Similar to the dosage effect above, sharp well-defined upstream zones would require greater than one-to-one *H* activation changes for a given shift in the *H-K* interface position (e.g., greater than 3-fold to induce a 3-fold positional shift).

*H-K dynamics can affect whorl radius and n_c_*

Experiments show that *H-K* mutual inhibition affects their interface position. The model indicates that, for observed *H*, *K* expression gradient steepness (corresponding to *n_H_* = 3), variation in inhibition [*H* or *K* concentrations (*prod*_ parameters), or inhibition strength (*inh*_ parameters), potentially mediated by small RNAs] can be close to proportional (one-to-one) with the induced positional shift.

Mechanism component variation for the conifer and *Arabidopsis* experiments involving *H-K* mutual inhibition are summarized in **Figure 9B**. Modelling experimentally increased inhibition or decreased production of HD-ZIP III generates the observed acropetal shifts. Increased inhibition or decreased production of *K* models the observed basipetal shifts. Notwithstanding differences working in non-model species, the shifts induced experimentally in *Arabidopsis* would be expected to work similarly in conifer embryos. The correlation between *n_c_* and whorl radius (*r*) and the wide range of *n_c_* in conifers (**Figure 3B**) provides a unique tool for future studies, allowing for monitoring positional shifts *via* the more readily obtained *n_c_*.

Overall increase or decrease in auxin levels (3.2.2.2.D) is likely the most readily transferable experiment from *Arabidopsis* to conifers. This could be a means for calibrating the perturbation-to-*n_c_* relation. Identifying observed shift magnitudes with those of model parameters (e.g. *prodH* or *P_0_*) could aid in corroborating the model, refining understanding of the interactions positioning the *H-K* interface. Altering HD-ZIP III or KAN levels in conifers would be expected to provide constraints on *prodH* and *prodK* values; and altering small RNA inhibitory strengths (e.g., miR166) could refine the *inh*_ parameters. Monitoring embryo radius could distinguish potential auxin growth effects from positional shifting.

In a fluctuating biochemical environment, the reliability of concentration-determined cell fate at expression zone boundaries is generally higher for sharp boundaries (short distances between ‘on’ and ‘off’ regions) than at more diffuse boundaries (shallower gradients). The modelling indicates that the reliability in cell fate corresponding to the observed sharp HD-ZIP III or KAN domains (Caggiano et al., 2017) could also be associated with a more stable position. The steeper the concentration gradient, the greater the required change in mechanism parameters to induce a particular positional shift (**Table 2**; Eq. 7; 3.2.1.2).

Conifer cotyledon patterning may therefore give insight into the balance of reliability and variability in development. Sharp domains of HD-ZIP III and KAN define a zone in which cells reliably have high auxin, in which primordia are initiated. However, the distance of this high auxin ring from the tip is variable, producing a variation in *n_c_* for the observed evenly-spaced cotyledons. In *Drosophila*, ~100 µm variation in *hb* expression domains has been studied as a means to establish reliable mid-embryo positions despite embryo size variability. Conifer cotyledon whorls may be displaying similar shifts in spatial pattering, potentially as a scaling to embryo size. The associated freedom in cotyledon number, though, suggests that *n_c_* is not under strong functional constraints in conifers. This contrasts with the consistent *n_c_* in monocots and dicots. In size also, variation appears more tightly regulated in the primordia initiation zone size of the SAM, where relatively small changes in radius are expected to alter phyllotaxy and therefore plant architecture (e.g. Jönsson et al., 2006, Figure 5). Future work in conifers and angiosperms will help to characterize the aspects of the primordia initiation zone size regulation mechanism responsible for the similarities and differences between species regarding developmental reproducibility and variability.

## Data availability statement

The original contributions presented in the study are included in the article/supplementary files. Further inquiries can be directed to the corresponding author.

## Author contributions

Experiments: RS and CW. Mathematics and computation: DH. Manuscript writing: DH and CW. All authors contributed to the article and approved the submitted version.
